# Relationship Quality and Cognition Across Race and Ethnicity

**DOI:** 10.1093/geroni/igaa057.2079

**Published:** 2020-12-16

**Authors:** Ji Hyun Lee, Indira Turney, Reza Amini, Benjamin Katz, Kristine Ajrouch, Toni Antonucci

**Affiliations:** 1 Florida State University College of Medicine, Tallahassee, Florida, United States; 2 Columbia University Medical Center, New York, New York, United States; 3 The University of Michigan-Flint, Flint, Michigan, United States; 4 Virginia Tech, Blacksburg, Virginia, United States; 5 Eastern Michigan University, Ypsilanti, Michigan, United States; 6 University of Michigan (UM), Ann Arbor, Michigan, United States

## Abstract

Quality of social relations have increasingly been recognized as an important factor in cognitive health in later adulthood. Less is known about the association of relationship quality with executive functioning (EF) and memory; and whether the links differ by race/ethnicity. In this paper, we investigated the associations between positive and negative quality of relationship with spouse, children, family, and friends with EF and memory across non-Hispanic Black, Hispanic, and non-Hispanic Whites. Participants are drawn from Health and Retirement Study Harmonized Cognitive Assessment Protocol (N = 2,678). Independent of network size and contact, relationship quality with family was linked to EF. Racial differences were found such that negative relationship quality with children was protective of EF for blacks. Relationship qualities were not associated with memory. These findings indicate that examining quality of relationship with distinct relationships may be essential for understanding the association with cognition, especially in the context of race/ethnicity.

